# Are both distinct epithelial and stromal cells molecular analysis from phyllodes tumors versus fibroadenoma components affected in breast fibroepithelial progression?

**DOI:** 10.1590/acb386823

**Published:** 2023-12-04

**Authors:** Ângela Flavia Logullo Waitzberg, Elisa Napolitano e Ferreira, Mabel Pinilla, Paulo Pineda, Andréa Cristina de Moraes Malinverni, Fernando Augusto Soares, Dirce Maria Carraro

**Affiliations:** 1 Universidade Federal de São Paulo – Paulista School of Medicine – Department of Pathology – São Paulo (SP), Brazil.; 2 Universidad de Concepción – Facultad de Medicina – Department of Medical Technology – Concepción, Chile.; 3 Hospital A C Camargo – Genomics and Molecular Biology Group – São Paulo (SP), Brazil.; 4 Universidade Federal de São Paulo – Laboratory of Molecular and Experimental Pathology I – São Paulo (SP), Brazil.; 5 Hospital A C Camargo – Department of Anatomic Pathology – São Paulo (SP), Brazil.

**Keywords:** Phyllodes Tumor, Fibroadenoma, Epithelial Cells, Stromal Cells

## Abstract

**Purpose:**

To determine molecular events involved in the tumorigenesis of phyllodes tumors (PT) and the role of each stromal (SC) and epithelial (EC) cell.

**Methods:**

Frozen breast samples enriched with epithelial and stromal cells from three fibroadenomas and 14 PT were retrieved and laser microdissected. Sanger and polymerase chain reaction-based sequencing of exon 2 MED12 and TERT promoter hotspot mutations were performed; 44K microarray platform was used to analyze gene expression.

**Results:**

All three fibroadenomas (FAs) presented mutations in *MED12*, but not in *TERT*, whose mutation was observed in five of the 14 PTs. EC and SC of each affected tumor displayed identical alterations. Of the total differentially expressed genes (DEG) (EC = 1,543 and SC = 850), 984 were EC-eDEGs and 291 were SC-eDEGs. We found a high similarity of diseases and functions enriched by both cell types, but dissimilarity in the number of enriched canonical pathways. Three signaling canonical pathways overlapping with EC and SC were predicted to be activated in one cell type and inactivated in the other, while no overlap in eDEGs was assigned to them. We also identified 13 EC-eDEGs and five SC-eDEGs enriched networks, in which the SC-eDEGs were able to segregate FA from PT samples.

**Conclusions:**

Identical TERT mutations from both SC and ES origins might affect the PTs tumorigenesis. Gene expression differences suggest coordinated molecular processes between these components with determinant differences acquired by SC, able to fully distinguish PTs from FAs lesions.

## Introduction

Phyllodes tumors (PT) are rare breast fibroepithelial lesions, accounting for < 1% of all breast tumors (Parker). PT can resemble fibroadenomas (FA) histology, but it is distinct from stromal hypercellularity^1^. Both breast tumors tend to have a benign behavior, although the former can be recurrent locally and undergo malignant progression to sarcoma^2^^,^ 3. Some evidence suggests that stromal cells (SC) may comprise the PTs neoplastic or transforming component^3^–^6^.

The histological analysis of stromal morphological features (i.e., mitotic index, overgrowth, cellularity, and atypia) defines the PT classification among benign, borderline, and malignant categories^7^^,^ 8. This classification does not fully match clinical outcomes, challenging to manage PT lesions. Histological similarities may further preclude distinguishing cellular FA from benign PT in some core biopsies and seem to involve common genetic features shared by these entities, such as *MED12* mutations^9^.

Despite the potential relevance of the stroma for PT development and progression, interactions between this component and the epithelium should not be ignored^10^^,^ 11. A preliminary evaluation displayed distinct chromosomal aberrations among the PT stroma (monoclonal) and epithelium (polyclonal)^12^^,^ 13. Nevertheless, the distinct role of epithelial-stromal interaction in this subset of tumors remains poorly understood.

This *MED12* gene [*OMIM 300188; cytogenetic band: Xq13.1; genomic location: chrX:71,118,543-71,144,103 (GRCh38/hg38)], protein called Mediator complex subunit 12, in addition to regulation, growth, and cell differentiation, participates in several signaling pathways. Another important gene is *TERT* [*OMIM 187270; cytogenetic band: 5p15.33; genomic location: chr5:1,253,147-1,295,068 (GRCh38/hg38)], which encodes the catalytic subunit telomerase, which in normal cells is responsible for the lengthening of telomeres and, when silenced, could be shortening. Thus, studies reveal that mutations in the *MED12* are more common in PTs. *MED12* mutations are more frequent in benign than malignant tumors, and in *TERT* they can lead to malignant progression^9^^,^ 11 ^,^ 14.

According to Pareja et al.^11^, two evolutionary pathways can be inferred, one of PTs with a previous history of FA and mutation in *MED12*, which will have a more favorable result, and another without pre-existing FA and regardless *MED12* mutation showed beneficial outcomes. In 2020, another study group demonstrated genetic mutations in both cell signaling pathways, tumor suppressor genes, DNA repair, and cell cycle regulatory pathways. In addition, they observed a high frequency of *MED12* mutation also recurrent mutations in *TP53*, *RARA*, and *PIK3CA* likely for tumor progression^9^.

New prognostic markers and therapeutic targets are claimed to improve the diagnosis, classification, outcome prediction, and management of PT-affected patients. Here, molecular differences between FA and PT were screened in both epithelial and stromal components of human biopsies, by sequencing the assessments displayed coordination of some molecular processes between epithelial (EC) and stromal (SC) cell types, including the *MED12* and *TERT* mutations, along with determinant differences acquired by SC that enabled us to fully distinguish PT and FA diseases.

## Methods

Fresh-frozen EC- and SC-enriched breast samples of three fibroadenomas and 14 phyllodes tumors surgically resected in 2015 and 2016 were retrieved from the A. C. Camargo Cancer Center Biobank, São Paulo, SP, Brazil. The donor cohort was composed of female patients (32–78 years old) with a nodular lesion diagnosed as fibroepithelial neoplasm and previously treated systemically. Cases were classified according to the latest World Health Organization criteria, reviewed by a pathologist with expertise in breast pathology (AFLW), and submitted to surgical excision (with margins, when necessary). In our PTs cases, six were considered benign or low-grade phyllodes tumors, and seven were considered borderline or malignant phyllodes, classified as high-grade cases. Data on the outcome and specific treatment schedule were obtained from clinical files. The protocol was approved by the Ethics Committee of the Medical and Research Center of A. C. Camargo Cancer Center (Protocol 1277/09).

### Nucleic acid extraction

**Figure 1:**
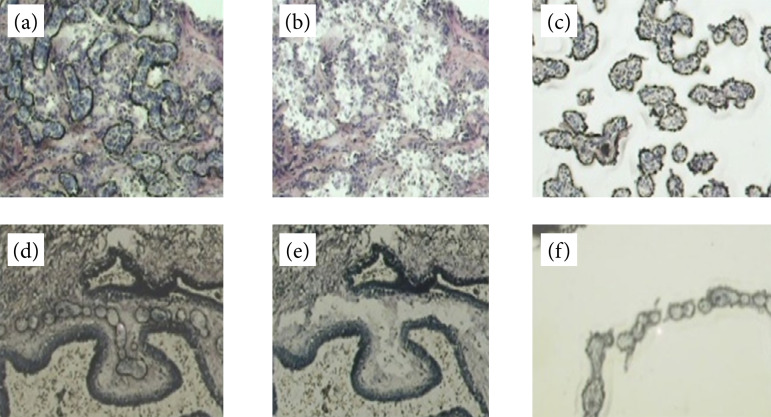
The sequence of microdissection in (a–c) epithelial and (d–f) stromal components. **(a)** Shooting epithelial cells; **(b)** same area without target cells; **(c)** equivalent slide with the target cells alone; **(d)** shooting stromal cells; **(e)** same area without target cells; **(f)** equivalent slide with the target cells alone.

Source: Elaborated by the authors.

The 4–8-μm thick sections of frozen samples were performed on Fisherbrand Superfrost glass slides (Thermo Fisher Scientific, Waltham, United States of America) for laser capture microdissection (LCM) of epithelial and stromal components on serial slides (Figs. 1a–1c and 1d–1f, respectively), on a Pixcell II LCM microscope (Thermo Fisher Scientific, Waltham, United States of America), using CapSure HS (Thermo Fisher Scientific, Waltham, United States of America) and a 7.5-μm diameter laser beam (maximum 10 min to avoid nucleic acid degradation). On average, 600 to 1,200 laser shots were performed to capture the target cells.

To isolate DNA, each slide was counterstained with staining solution (Histogen–Arcturus) for 30 seconds, followed by dehydration in alcohol (75%, 30 seconds; 95%, 30 seconds; 100%, 1 minute) and xylene. According to the manufacturer’s recommendations, DNA was extracted and purified using the QIAamp DNA Micro Kit (Qiagen). DNA quantity and quality were evaluated by spectrophotometry (Nanodrop 2000, Thermo Fisher Scientific) and 0.8% agarose gel electrophoresis.

To isolate the RNA, each slide was treated with 75% ethanol (1 minute) and distilled water (30 seconds), stained with histogenic staining solution (Thermo Fisher Scientific, Waltham, United States of America) for 30 seconds, and dehydrated in alcohol (75%, 30 seconds; 95%, 30 seconds; 100%, 1 minute) and xylene. Total RNA was extracted using the PicoPure RNA Isolation Kit (Thermo Fisher Scientific, Waltham, United States of America) and the RNase-Free DNase Set (Qiagen, Germantown, United States of America) for DNase treatment. RNA quality was assessed on a bioanalyzer 2100 (Agilent, Santa Clara, United States of America) using the Pico LabChip RNA 6000 kit (Agilent, Santa Clara, United States of America). All these procedures were performed according to the manufacturer’s recommendations.

### Sequencing for MED12 and TERTp mutations

Mutations in the exon 2 of MED12 and TERTp (C228T and C250T) were screened by Sanger sequencing (SS), after its amplification with the specific primers (Table 1). Q-Solution (Qiagen) was added to the reaction to amplify both exon 2 of MED12 and TERTp by polymerase chain reaction (PCR), and the PCR products were purified in agarose gel electrophoresis labeled with Big Dye Terminator (Applied Biosystems, Foster City, CA, United States of America), with bidirectional primers, and subjected to 3130 × l Genetic Analyzer (Applied Biosystems, Foster City, CA, United States of America), by standard protocols. The Catalogue of Somatic Mutations in Cancer (COSMIC) database 48 was used to identify already-known somatic mutations and mutation types.

**Table 1:** Primer sequences to mutations in the exon 2 of *MED12* and *TERTp* mutations.

Gene	Primer sequence
*MED12*	Exon 2 forward 5’-AACTAAACGCCGCTTTCCTG-3’
	Exon 2 reverse 5’-TTCCTTCAGCCTGGCAGAG-3’
*TERTp*	Promoter forward 5’-AGCGCTGCCTGAAACTCG-3’
	Promoter reverse 5’-CCTGCCCCTTCACCTTCCAG-3’

Source: Elaborated by the authors.

### Global gene expression

#### mRNA amplification and labelling

Total RNA was submitted to a two-round linear amplification procedure, based on a T7-driven methodology^15^, and the RNA was labeled with a Low Input Quick Amp Labeling kit (Agilent Technologies), according to the manufacturer’s recommendations. Each tumor sample was labeled with Cy3-CTP, and a reference sample was labeled with Cy5-CTP. A “spike A” labeled with Cy3-CTP and a “spike B” labeled with Cy5-CTP served as controls.

#### Microarray

The Whole Human Genome Oligo Microarray 44K (Agilent Technologies), comprising 44,074 probes for one or more exons from all coding genes of known human proteins, was used, and the reference sample was performed on the same slide^16^. The Cy3-RNA sample (450 ng) was added to the same amount of reference Cy5-RNA and hybridized (at least 20 hours, 65°C) with 10 rpm, with buffer Hi-RPM (Agilent, United States of America). The background was eliminated by further washing process (Agilent, United States of America) and drying with acetonitrile for 30 seconds. Arrays were scanned at Scanner Agilent Bundle Model B (Agilent, United States of America) with two lasers that stimulate Cy3 or Cy5 fluorophores. Intensity values from all scanned spots were extracted and processed by the Agilent Feature Extraction program (version 10.7.1), with fluorescence intensity of all transcripts and control references extracted with correction factors for each Agilent slide.

#### Data processing and analysis

**Figure 2:**
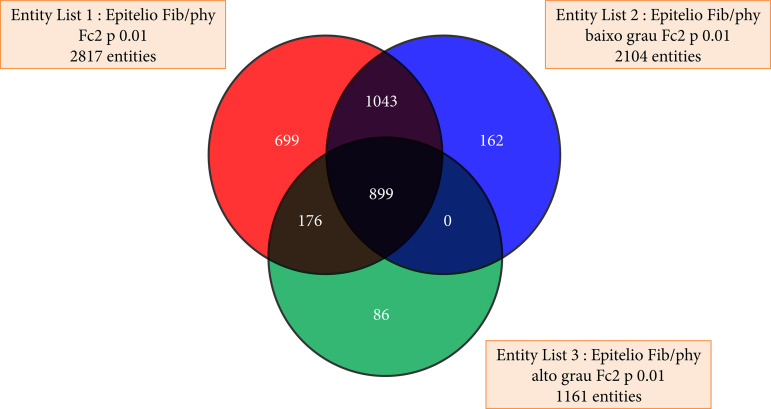
Venn diagram all probes.

Source: Elaborated by the authors.

Differentially expressed genes (DEGs) were selected based on fold change ≥ |2| and p < 0.01. Outlier probes and those matching blank gene symbols were not evaluated. A Venn diagram (Fig. 2) was used to identify the DEGs between EC and SC that were exclusive of only one cell type.

For clustering analysis, *e*xpression values were Z-score transformed and submitted to an unsupervised hierarchical clustering with Euclidean distance and average linkage using MeV software (Multiple Experiment Viewer, Version 4.4.1). The reliability of clustering was assessed by bootstrapping (100 iterations). Genes with a similar expression profile among samples were identified by visual inspection and were excluded from further analysis.

For functional enrichment analysis of DEGs between FAs and PTs, we performed a core analysis of the ingenuity pathway analysis (IPA) system (Qiagen, Germantown, United States of America). Default parameters (IKB reference gene population; direct and indirect gene interactions; molecules per network ≤ 35; networks per analysis ≤ 25; experimental evidence for statistical significance; P < 0.05) were used to identify the enrichment of diseases and disorders, molecular and cellular functions, gene interaction networks and canonical pathways with their predicted activated or inactivated status.

## Results

### Somatic mutations

A total of five (29.4%) cases showed only MED12 mutations, including all three FAs. Mutations were identical in SC and EC, described as c.131G > C; p.Gly44Ala(297271), c.131G > A; p.Gly44Asp, (1119981) and c.107_110inv; p.(Leu36_Thr37delinsArgGln)(745491). All the MED12 mutations were found at the second codon, but not in the same loci, with the codons 129, 130, and 131 also showing changes. A total of two (11.8%) cases had only TERT mutations. All cases with TERT mutations showed the same spot (124) and were identical in EC and SC. Simultaneous TERT and MED12 alterations were present in three (17.6%) cases, while both genes were preserved in seven (41.2%) cases. Unlikely the observed for MED12, TERT alterations were not observed in any of the three FAs, but occurred in five of the 14 PTs. The type of alterations matched some present in PTs samples (codon 131) and was identical in SC and EC (Table 2).

**Table 2:** Sanger sequencing: mutation analysis in the *MED12* gene*.

Case	Samples	Epithelial component	Stromal component
**Wild**			
1	M 474T-Epi	Wild	Wild
2	M 476T-Epi	Wild	Wild
3	M 477T-Epi	Wild	Wild
4	M 478T-Epi	Wild	Wild
5	M 480T-Epi	Wild	Wild
6	M 481T-Epi	Wild	Wild
7	M 483T-Epi	Wild	Wild
**Mutation**			
8	M 479T-Epi	c. 131 G > T; p. Gli44Cis	c. 131 G > T; p. Gli44Cis
9	M 486T-Epi	c. 130 G > T; p. Gli44Cis	Wild
10	M 487T-Epi	c. 130 G > T; p. Gli44Cis	c. 130 G > T; p. Gli44Cis
11	M 488T-Epi	c. 131 G > C; p. Gli44Ala	c. 131 G > C; p. Gli44Ala
12	M 489T-Epi	c. 131 G > A; p. Gli44Asp	c. 131 G > A; p. Gli44Asp
13	M 490T-Epi	c. 107_110invGTCA; p. Leu36_Thr37delinsArgGln	c. 107_110invGTCA; p. Leu36_Thr37delinsArgGln

* Mutation rate: 6/13 = 50%; mutation in both cell types: 5/6 = 83%. Source: Elaborated by the authors.

### Gene expression profiles

The mRNA was obtained from 16 (13 PTs and three FAs) of the 17 cases to analyze microarray analysis. First, we found almost twice more DEGs in EC than in SC (fold change ≥ |2|; P < 0.01), but no significant difference was observed in the number of upregulated or downregulated genes among cell types and fibroepithelial lesions. Next, exclusive differentially expressed genes (eDEGs) of EC (EC-eDEGs) and SC (SC-eDEGs) samples were identified, and more eDEGs were observed in EC than in SC. However, this analysis showed SC harboring much more upregulated genes in PT than in FA when compared to epithelial cells: a total of 984 upregulated EC-eDEGs (FA = 412 and PT = 572) and 291 upregulated SC-eDEGs (FA = 61 and PT = 230) were identified.

### Clustering analysis

**Figure 3:**
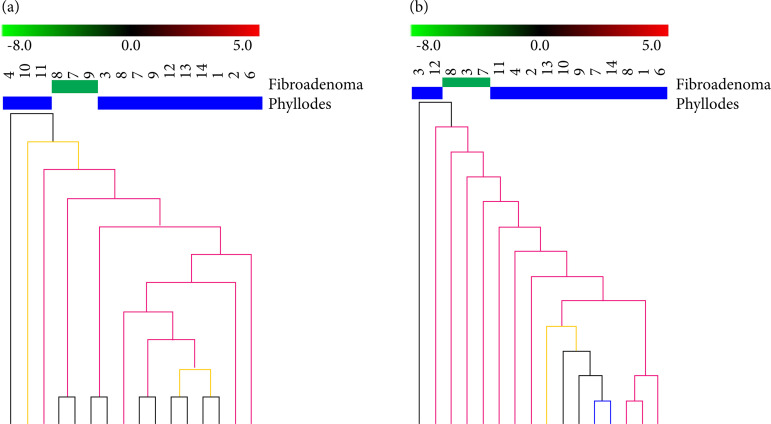
Hierarchical clustering analysis of samples based on differentially expressed genes between phyllodes tumors and fibroadenomas for each component. **(a)** Epithelial component. **(b)** Stromal component.

Source: Elaborated by the authors.

To evaluate the ability of the expression profile based on the 984 EC-eDEGs and 291 SC-eDEGs to correctly discriminate the FA from PT samples, we performed an unsupervised hierarchical clustering analysis with Euclidean distance and average linkage of 16 EC samples (three FAs and 13 PTs) and of 16 SC samples (three FAs and 13 PTs) (Fig. 3). The EC-eDEGs profile distinguished two (66%) FA samples from 13 (100%) PT samples. More interestingly, the SC-eDEGs profile distinguished three (100%) FA samples from 13 (100%) PT samples. The reliability of clustering was assessed by bootstrapping (100 iterations). These findings suggest SCharbor molecular differences that would be more determinant of PT tumorigenesis.

### Phyllodes tumors

#### Biological function analysis

According to our IPA analysis, EC-eDEGs and SC-eDEGs were enriched for similar top diseases and functions. In this context, sought to explore some functional differences between EC and SC pathways and identified an overlap of five enriched canonical pathways, of which three were predicted to be activated in one cell type and inactivated in the other one (inverted activation status). Interestingly, there was no overlap of the EC-eDEGs and SC-eDEGs that were enriched for the three canonical pathways with inverted activation status. Next, by interrogating the interconnection among EC-eDEGs and four SC-eDEGs, it was performed a functional network enrichment analysis and found that EC-eDEGs were enriched for 13 functional networks (score ≥ 35). In addition, it was found that SC-eDEGs were enriched for five functional networks (score ≥ 35). Diseases and functions belonging to more than one network were assigned hierarchically, following descending order of occurrence.

## Discussion

FAs and PTs share some morphological overlapping features and FAs-like areas which are rarely found in PTs^17^, challenging the diagnosis of some cases. Several studies have focused on screening changes in the gene PTs pattern, aiming to better distinguish it from benign FAs for diagnosis. Recurrent mutations affecting exon 2 of MED12, a gene codifying a protein involved in the transcriptional regulation of gene expression, are widely observed in both FAs and PT^18^. Frequent genetic mutations in TERT promoter and RARA in both these breast tumors are also reported^19^.

This study found a prevalence of MED12 mutations in benign FAs (100%) than in PTs (11.8%) and the presence of TERT mutations only in PTs (36%). These findings do not fully support MED12 mutations as a common and early pathological event in these fibroepithelial tumors, as did previous studies showing a high frequency and similar patterns of MED12 mutations in both FAs and various grades of PTs^20^–^22^. On the other hand, they quite support a previous study suggesting a role of TERT mutations on the development of these breast tumors and in segregating them from FAs, as it was frequent in PTs (30/46, 65%) and rare in FAs (4/58, 7%)^21^^,^ 23.

More relevantly, contrasting to previous studies showing confinement of MED12^17^^,^ 21 and TERT^24^ mutations in the stromal component of PTs, all the mutations in both MED12 and TERT identified had identical EC and SC contributions. Our data support the hypothesis that epithelial-stromal interactions affect the development of fibroepithelial lesions. This hypothesis is based on an initial observation of a tendency of the stromal mitotic activity of these tumors to occur near the epithelial compartment, suggesting that stromal growth in these tumors depends (at least in part) on the epithelial component^25^. A number of studies exploring this hypothesis further reported allelic chromosomal imbalances^26^ and clonal abnormalities^27^^,^ 28 in both epithelial and stromal elements of PTs, abnormalities in pathways for stromal proliferation raising from the epithelial component in benign PTs^29^, as well as correlations of increasing malignancy with a loss of stromal dependency on the epithelium^25^^,^ 29 and of epithelial E-cadherin expression with recurrence and shorter tumor specific survival in non-benign PTs^30^.

Accordingly, contrary to the general morphological impression, the preliminary evaluation of DEGs encountered in our study indicated that both EC and SC might be affected in FAs and PTs. Furthermore, although PTs are considered fibroepithelial lesions with increased stromal^17^ and often exhibit a morphologically preserved epithelial component, epithelial results were numerically higher than stromal component DEGs numbers. While we found 1,502 DEGs with 864 upregulated genes in epithelial microdissected cells, stromal component evaluation resulted in 890 DEGs with 526 upregulated genes.

Some subtle changings in epithelial gene expression could be explained by paracrine epithelial-mesenchymal interaction, but the finding of epithelial DEGs higher than stromal components can raise several other hypotheses, because the epithelium is compromised by stromal hyperplasia and transformation adaptively. According to a large recent review, scarce gene alterations apart from MED-12 mutations have been reported in FAs^31^. Since PTs were compared to FAs, the genetic upregulation phenomena should be regarded as PTs alterations. Another hypothesis is that alterations in the epithelial component are then transitory or not oncogenic per se, being these cells more prone to gene imbalance or adaptation to adverse scenarios. Comparing the gene profile alterations in invasive carcinoma (epithelial origin) to PTs (stromal-related transformation), Tan et al.^17^ found distinctive profiles of mutated genes, suggesting that epithelial and stromal origins are related to different drivers’ mutations and carcinogenic processes.

In this study, *MED12* and *TERT* were identical in the two components, and it must suggest that they come from a common origin, and our microarray results also indicated the same event. Although *MED12* is not useful as a prognostic factor or a potential marker for FAs or PTs, in accordance with previous findings, *MED12* exon 2 mutation, when detected, could help distinguish PTs from other spindle neoplasms of the breast, such as myofibroblastomas, nodular fasciitis, and sarcomas, which is also a common scenario in breast pathology when it faces a stromal lesion^20^.

On the other hand, *TERT* promoter mutations were rare in FAs and, according to literature, are progressively more frequent in benign, borderline, and malignant categories of phyllodes tumors. Moreover, *TERT* promoter mutations are the most frequent, occurring in up to 70% of malignant/borderline PTs and found in 50% of benign PTs^21^^,^ 24. Therefore, one hypothesis already mentioned is that, since *TERT* mutations are rare in FAs (0–7%), these mutations could drive the progression of PTs from FAs^14^. It has also been suggested that *TERT* mutations may be useful in distinguishing between benign PTs and cellular FAs, when present^17^.

Interestingly, the pathways involved showed high similarity in those exclusive DEGs of both EC and SC, indicating that the same cellular functions are frequently affected in both components. This feature of alterations could infer a common source of SC alteration delivering common genetic alterations to both components in an early carcinogenic phase. Furthermore, eDEGs from SC were able to fully distinguish PTs from FAs, meaning that SC contained gene expression features highly like FAs, but the opposite was not observed. In addition to the observed broad number of genes differentially expressed in PTs when compared to FAs, which may reflect the progression of mesenchymal carcinogenesis, this finding can support a hypothesis that considers FAs as PTs precursors. This hypothesis is based on the non-rare presence of histological FA-like areas in PTs and is reinforced by molecular evidence of the loss of heterozygosity and human androgen receptors^32^^,^ 33.

The main limitations of our study are its short sample size and the control adopted. Although within a limited number of samples, in our series *TERT* mutations were present in borderline and malignant PTs. However, one case of malignant PT did not show any *TERT* mutations. When we closed the results, the five-year follow-up available of all patients did not show any recurrence so the presence of *TERT* mutation, apparently, did not drive a worse diagnosis. The ideal control for our study should be EC and SC from normal mammary tissue. However, since the amount of EC and myoepithelial cells are much higher than SC, our tentative to sample adequate SC from normal mammary tissue was frustrated by scarce cellularity and poor RNA content. Therefore, we decided to compare mesenchymal cells from PTs to those from FAs, surely also composed of benign hyperplastic SC. Although FAs may also contain hyperplastic or neoplastic epithelial lesions, we collected juvenile FAs with previously known benign morphology by reviewing the hematoxylin and eosin diagnostic slides. It is worth noting that these limitations do not affect the main contribution of our study, showing a very similar contribution of EC and SC on molecular changes of PTs and FAs.

## Conclusion

Identical MED12 and TERT mutations were observed in both SC and EC and the former might affect the PTs tumorigenesis. Gene expression differences suggest coordinated molecular processes between these components with determinant differences acquired by SC, able to fully distinguish PTs from FAs. A comparison of the SC gene expression pattern between these breast tumors suggests FAs as PTs precursors.

## Data Availability

The data will be available upon request
